# Screening for Psychological Distress in Adult Primary Brain Tumor Patients and Caregivers: Considerations for Cancer Care Coordination

**DOI:** 10.3389/fonc.2015.00203

**Published:** 2015-09-22

**Authors:** Wafa Trad, Eng-Siew Koh, Maysaa Daher, Alanah Bailey, Marina Kastelan, Dianne Legge, Marcia Fleet, Grahame K. Simpson, Elizabeth Hovey

**Affiliations:** ^1^Liverpool Cancer Therapy Centre, Liverpool Hospital, Sydney, NSW, Australia; ^2^University of New South Wales, Sydney, NSW, Australia; ^3^Ingham Institute for Applied Medical Research, Sydney, NSW, Australia; ^4^Liverpool Brain Injury Rehabilitation Unit, Liverpool Hospital, Sydney, NSW, Australia; ^5^Prince of Wales Hospital, Sydney, NSW, Australia; ^6^Sydney Neuro-Oncology Group, North Shore Private Hospital, Sydney, NSW, Australia; ^7^Northern Sydney Cancer Centre, Royal North Shore Hospital, Sydney, NSW, Australia; ^8^Olivia Newton-John Cancer & Wellness Centre, Austin Health, Heidelberg, VIC, Australia; ^9^Department of Neurosurgery and Medical Oncology, Royal Melbourne Hospital, Parkville, VIC, Australia; ^10^Department of Medical Oncology, Prince of Wales Hospital, Sydney, NSW, Australia

**Keywords:** primary brain tumor, screening, psychological distress, neuro-oncology, care coordination

## Abstract

**Introduction:**

This study aimed to assess psychological distress (PD) as scored by the Distress Thermometer (DT) in adult primary brain tumor patients and caregivers (CGs) in a clinic setting and ascertain if any high-risk subgroups for PD exist.

**Material and methods:**

From May 2012 to August 2013, *n* = 96 patients and *n* = 32 CG underwent DT screening at diagnosis, and a differing cohort of *n* = 12 patients and *n* = 14 CGs at first recurrence. Groups were described by diagnosis (high grade, low grade, and benign) and English versus non English speaking. Those with DT score ≥4 met caseness criteria for referral to psycho-oncology services. One-way ANOVA tests were conducted to test for between-group differences where appropriate.

**Results:**

At diagnosis and first recurrence, 37.5 and 75.0% (respectively) of patients had DT scores above the cutoff for distress. At diagnosis, 78.1% of CGs met caseness criteria for distress. All CGs at recurrence met distress criterion. Patients with high-grade glioma had significantly higher scores than those with a benign tumor. For patients at diagnosis, non English speaking participants did not report significantly higher DT scores than English speaking participants.

**Discussion:**

Psychological distress is particularly elevated in CGs and in patients with high-grade glioma at diagnosis. Effective PD screening, triage, and referral by skilled care coordinators are vital to enable timely needs assessment, psychological support, and effective intervention.

## Introduction

Patients with primary brain tumors (PBT) experience a myriad of complex physical, emotional, cognitive needs (1, 2), which can have adverse psychological effects on both the patient and their caregivers. In particular, patients diagnosed with high-grade glioma (HGG) have a poor prognosis and limited life expectancy and as such often experience rapid decline in physical and cognitive functioning (3). They can often exhibit a dynamic constellation of needs throughout the care trajectory, from initial diagnosis to tumor recurrence through to the palliative phase (4, 5), with profound changes not only in physical functioning but also in psychological distress (6), mood, cognition, and behavior.

Due to the high emotional sequelae of having a PBT (7), it is important that patients are routinely screened for psychological distress and have effective and timely interventions instituted. The National Comprehensive Cancer Network (NCCN) Distress Thermometer (DT) is one of the most familiar and widely adopted psychological distress screening tools utilized in oncology populations (8, 9). Several studies have described DT screening in malignant and benign PBT cohorts, with the majority undertaking assessments relatively soon after initial diagnosis. Keir et al. (10) assessed a convenience sample of malignant PBT patients with time interval from tumor diagnosis to testing varying from 10 days to 22 years, with a median time of 1.8 years. Using a cutoff score of DT ≥4, 52% of their cohort were classified as suffering from elevated psychological distress. Similarly, Kvale et al. (11) applied the DT in a sample of 50 glioblastoma patients, with 22% of patients seen within the first 4 months after diagnosis. They reported a mean distress score of 2.15 (SD = 2.66), with caseness criteria using a DT score of ≥4 met by 28%.

Only a few studies have published serial or longitudinal DT data in PBT populations; however, assessment was acquired at pre-defined chronological time points such as baseline, and six monthly (12), or after a short interval such as pre- and post-radiotherapy (13). These time points did not specifically reflect the actual treatment phase or disease status such as at tumor recurrence (14) or the terminal phase when distress is known to be elevated (4, 15). Keir et al. (6) categorized 83 glioblastoma patients into those who had been diagnosed either less than or greater than 18 months. They reported that 59% of long-term survivors (LTS) met the DT ≥4 cutoff score for distress (M = 4.61, SD = 3.12) compared with 49% of those diagnosed <18 months.

It is widely acknowledged that the impact and burden on caregivers of brain tumor patients is significant (3, 16–19). Caregivers can experience a range of unmet supportive care needs and remain at risk of physical, emotional, and financial stressors themselves (2, 20–22). Despite this, there is currently only sparse literature documenting both patient and caregiver psychological distress levels.

It is notable that even in subgroups of PBT survivors with more favorable outcomes such as pituitary tumors (23) or low-grade glioma (LGG) (24), a spectrum of concerns including neurocognitive and behavioral issues (25) and elevated psychological distress has been detected (13). PBT patients and caregivers are faced with a new reality of living over months to even years with the sequelae of brain tumor, due to the tumor and/or the effects of therapy (26). Furthermore, it is likely that the existing language barriers, the knowledge and ability to navigate through a complex healthcare system, cultural insensitivities, and a lack of patient-centered care (27, 28) affecting Culturally and Linguistically Diverse (CALD) cancer groups (29), are likely to be heightened in those patients and caregivers faced with the diagnosis of PBT (30).

The complexity and scope of needs experienced by brain tumor patients and their caregivers requires a coordinated response from healthcare services and providers. In Australia, a cancer care coordinator is a position focused specifically on improving the patient journey. The care coordination role is designed to incorporate the critical functions of assessment and evaluation of clinical and supportive care needs and liaising with multidisciplinary teams (MDTs) to achieve timely and high quality care (31, 32).

This study aimed to assess the psychological distress of PBT patients and caregivers, as measured by the DT, in a clinic setting. The second aim was to ascertain if any high risk subgroups for psychological distress exist, for example, caregivers and those of non English speaking background (NESB).

## Materials and Methods

### Patient population

The study population comprised PBT patients who were either newly diagnosed or who were experiencing first tumor recurrence. Likewise, as a separate group, caregivers of PBT patients with either malignant or benign tumors were assessed and underwent DT screening from the period of May 2012 to August 2013. PBT categories included HGG, LGG, and benign brain tumors (BBTs), the most common of which included meningioma and pituitary tumors.

### Neuro-oncology care coordinator role in south-west sydney

The NOCC in South Western Sydney Local Health District (LHD), serving a population of almost one million people, is a one full-time equivalent position that encompassed the care of all primary malignant and benign brain and spinal tumor patients diagnosed across three main teaching hospitals in the LHD. New cases and referrals were identified at MDT meetings held every 2 weeks where all new and recurrent PBT cases were discussed with a consensus management plan recommended.

### Psychological distress screening

#### Study Procedures

Ethical approval to undertake the study was provided by South Western Sydney Area Health Service Human Research Ethics Committee. The NOCC conducted a screening assessment with each participant using the DT tool for psychological distress screening. DT screening was performed around the time of initial PBT diagnosis or at the time of first tumor recurrence. Likewise, DT screening was undertaken in caregivers of PBT patients either at initial diagnosis or first recurrence. DT screening for non English speaking patients was administered in the presence of a hospital-based interpreter where available or alternatively an English speaking caregiver. The majority of DT screening assessments were performed with the NOCC face-to-face in an oncology outpatient setting, or alternatively by phone, especially if patients were too unwell to attend clinic or had other logistical challenges.

#### Study Measures

The DT, a measure (8, 9, 33) validated in a variety of multisite cancer populations, was used to screen for psychological distress in the study cohort. Participants rated their level of distress in the past week on a scale of 1–10, with 0 = no distress, 5 = moderate distress, and 10 = extreme distress. Patients were then asked to indicate areas of problems listed under the following five main categories: physical, family, emotional, spiritual/religious, and practical. Patients and/or caregivers were subsequently referred onto relevant psycho-oncology services for further evaluation and intervention according to the problems identified.

The DT has traditionally been designed for and utilized in cancer patients only (33). However, it is widely acknowledged that there are multiple identified supportive care needs that exist in PBT caregivers (1, 3, 34). In addition, Zwahlen et al. reported a validation study supporting the use of the DT in screening the caregivers of cancer patients (35). Hence, where feasible in the current study, caregivers were also asked to complete the DT.

Patients and/or caregivers with a DT score ≥4 were referred by the NOCC to relevant psycho-oncology, Allied Health or community services, in keeping with documented and accepted clinical practice guidelines for psychological distress management in oncology (9).

Selected patient demographic data including age and country of origin, English speaking versus non English speaking background, as well as clinical data including the PBT diagnosis, nature/timing of relevant treatments, and date of first (but not subsequent) tumor recurrence were collected.

It is widely acknowledged that there are many differing definitions for CALD groups. Although the Australian Bureau of Statistics (36) utilizes the category of “people who were born overseas,” for the purposes of the current study and also for the subsequent analysis, patients and caregivers were grouped into English speaking versus non English speaking groups rather than by country or regions of birth.

### Data management and statistical analyses

The oncology electronic medical record, Mosaiq^®^, was used as a platform to collect and extract relevant clinical information concerning all DT scores from patients and caregivers. Once extracted from Mosaiq^®^, descriptive statistics were generated for all variables.

Results have been ordered according to four independent participant groups: patients at diagnosis, patients at recurrence, caregivers at diagnosis, and caregivers at recurrence. Descriptive and frequency data were generated for DT scores. Next, for patients at diagnosis, an independent samples *t*-test tested for differences on DT scores between English speaking and non English speaking background participants. Then, a one-way ANOVA was conducted to test for statistical differences among the three tumor diagnosis groups (HGG versus BBT versus LGG). Scheffe *post hoc* comparisons were then conducted with an adjusted Bonferroni alpha of *p* < 0.016 (0.05/3). Due to insufficient sample size, similar tests were not conducted for any of the other three participant groups. Finally, scores on the DT were divided into two groups (<4 and ≥4) to identify caseness. Caseness can be defined in this context as any participant with psychological distress levels that were clinically significant, i.e., that require further assessment and/or intervention by psycho-oncology services. Participants with DT scores ≥4 were classified as meeting caseness criteria (8).

## Results

### Sample

In total, 190 DT scores were collected. Of these, 25 patients and 11 caregivers had completed the DT at more than one time point. In these cases, only the first instance of DT completion was retained for analysis. Data from a total of 154 DT scores remained. DT scores were collected from a varied neuro-oncology cohort that comprised HGG, LGG, and BBT patients, as categorized by the international WHO 2007 classification of brain tumors (37). All patients had a confirmed histopathological diagnosis. LGG patients were those with grade I or II glioma in contrast to those with BBT who were predominantly patients with meningioma and pituitary tumors.

Of the total number of DT assessments, 30.5% (47/154) were performed in NESB participants. Table 1 provides a summary of DT scores for PBT patients and caregivers, both at diagnosis and first tumor recurrence, and according to tumor subtypes and English speaking background.

**Table 1:** **Summary of distress thermometer scores for primary brain tumor patients and caregivers, both at diagnosis and first tumor recurrence, according to tumor subgroups and English speaking background**.

	Patient		Caregiver	
	Diagnosis (*n* = 96)	Recurrence (*n* = 12)	Diagnosis (*n* = 32)	Recurrence (*n* = 14)
**Total**				
Mean, SD	3.15 ± 2.20	5.42 ± 3.09	5.34 ± 1.89	7.64 ± 1.50
Range	0–8	1–10	2–8	5–10
**HGG**				
*n*, %	39 (40.6)	8 (66.7)	22 (68.8)	11 (78.6)
Mean, SD	4.03 ± 2.36	5.13 ± 2.98	5.86 ± 1.81	7.64 ± 1.29
Range	0–8	1–9	3–8	5–9
**LGG**				
*n*, %	8 (8.3)	1 (8.3)	4 (12.5)	1 (7.1)
Mean, SD	2.25 ± 1.28	2.00	4.25 ± 1.71	8.00
Range	0–4	–	2–6	–
**BBT**				
*n*, %	49 (51.0)	3 (25.0)	6 (18.8)	2 (14.3)
Mean, SD	2.59 ± 1.97	7.33 ± 3.06	4.17 ± 1.72	7.50 ± 3.54
Range	0–8	4–10	2–7	5–10
**ESB**				
*n*, %	69 (71.9)	9 (75.0)	19 (59.4)	10 (71.4)
Mean, SD	3.03 ± 2.26	5.11 ± 2.67	5.53 ± 1.87	7.40 ± 1.43
Range	0–8	1–9	2–8	5–9
**NESB**				
*n*, %	27 (28.1)	3 (25.0)	13 (40.6)	4 (28.6)
Mean, SD	3.44 ± 2.06	6.33 ± 4.73	5.08 ± 1.98	8.25 ± 1.71
Range	0–7	1–10	3–8	6–10

*HGG, high-grade glioma; LGG, low-grade glioma; BBT, benign brain tumor; ESB, English speaking background; NESB, non English speaking background*.

### Patients

A total of 96 DT scores were collected from patients at diagnosis, comprising 40.6% (39/96) with a diagnosis of HGG, 8.3% (8/96) with LGG, and 51.0 (49/96) with BBT. The highest mean DT scores were those with HGG tumors, followed by BBT and then LGG. For patients at diagnosis, 28.1% (27/96) were from NESB participants. DT scores were similar among NESB participants and those from an English speaking background (Table 1). Of all patients at the time of diagnosis, 37.5% (36/96) met criteria for caseness using the DT.

An independent samples *t*-test found no significant differences on DT scores between those from an English speaking and non English speaking background. A one-way ANOVA found statistically significant differences on DT scores among the three tumor groups, *F*(2, 93) = 5.882, *p* < 0.05. Scheffe *post hoc* tests showed that scores on the DT for the HGG diagnosis group were significantly higher than those with a diagnosis of BBT (*p* < 0.016). No other significant differences were found.

Twelve patients at recurrence completed the DT. Of these, 66% (8/12) had a diagnosis of HGG, 8.3% (1/12) had LGG, and 25.0% (3/12) had BBT. Those with a diagnosis of BBT had the highest scores on the DT. Similar to patients at diagnosis, 25.0% (3/12) of patients at recurrence were from an NESB; however, scores on the DT were slightly elevated in comparison with those from an English speaking background. The majority of patients at recurrence met caseness criteria using the DT (75.0%, 9/12).

### Caregivers

A total of 32 caregivers at diagnosis completed the DT. These comprised 68.8% (22/32) supporting someone with HGG, 12.5% (4/32) with LGG, and 18.8% (6/32) with BBT. The highest DT scores were for those with a diagnosis of HGG. There was a high rate of caregivers at diagnosis from an NESB (40.6%, 13/32), and they had slightly higher DT scores than those from an English speaking background (Table 1). A large proportion of caregivers at the time of diagnosis met the caseness criteria for the DT (78.1%, 25/32).

Finally, DT scores were collected from 14 caregivers at recurrence. These were composed of 78.6% (11/14) supporting someone with HGG, 7.1% (1/14) LGG, and 14.3% (2/14) BBT. Scores on the DT were high among all tumor gradings for caregivers at recurrence, although the small group numbers did not allow for further comparison. Just under 30% of caregivers at recurrence were from an NESB (28.6%, 4/14) with similar mean scores on the DT (Table 1). All caregivers completing the DT at first recurrence had scores which met caseness criteria (100%, 14/14).

## Discussion

Primary brain tumor patients have complex supportive care needs, and caregiver burden is high (2, 3, 21). At initial diagnosis, the poor prognosis associated with HGG, coupled with physical, neurocognitive, and behavioral sequelae associated with the disease and its associated therapies, can contribute to elevated psychological distress. Furthermore, due to the almost certain pattern of recurrence in HGG over time leading to high mortality rates, psychological distress levels can potentially increase throughout the disease journey.

In the current study, 37.5% of all newly diagnosed patients in the current population met caseness criteria for psychological distress using the DT. This is comparable to other published literature where, using the same cutoff value, caseness ranged from 28% in newly diagnosed patients with glioblastoma with mean distress score of 2.15 (SD = 2.66) (11) to 52% in another PBT cohort at diagnosis (10). Similarly, Goebel et al. (38) assessed 159 patients with varied types of malignant and benign PBT, the majority of whom were diagnosed within the last 3 months. Using a higher DT cutoff of ≥6, 48.4% of patients were experiencing distress with 6.9 sources of cancer-related distress. DT scores were significantly associated with depression and anxiety as well as the reported number of concerns.

The findings of the current study confirm that levels of psychological distress as measured by the DT are high in patients with PBT. In patients overall, the DT score a mean of 3.15 (SD, 2.20) at initial diagnosis and a mean of 6.49 (SD, 2.61) at first tumor recurrence. In comparison, distress was higher in caregivers overall, with mean DT score at diagnosis of 5.34 (SD, 1.89) and 8.2 (SD, 1.47) in caregivers assessed at first recurrence.

The results presented here also highlight that psychological distress was particularly elevated in patients with HGG compared with low grade and benign tumor groups overall. Such a finding is intuitive, given the propensity for relatively rapid tumor recurrence and progressive functional decline, and is supported by other studies with similar conclusions (10, 11). Given the cross-sectional design of the current study and the small numbers of patient–caregivers dyads who were assessed with the DT at both diagnosis and again at first recurrence, it is not possible to make any conclusions about the longitudinal patterns of psychological distress.

There are a number of published studies that have attempted to describe the longitudinal patterns of psychological distress and/or mood disorder. Rooney et al. (12) sampled newly diagnosed glioma patients at three time points: T1 (*n* = 154 patients) shortly after starting chemo/radiotherapy, T2 (*n* = 103 patients) 3 months later, and T3 (*n* = 83 patients) 6 months later. Significant distress was present in 36.4% at T1, 35.9% at T2, and 33.7% at T3. Longitudinally, subjects with high distress at T1 (median DT score = 8) remained highly distressed at follow-up (T2 median = 8, T3 median = 7). Younger age, functional impairment, and concurrent major depressive disorder were independently associated with high distress with emotional difficulties among the most common causes of distress at all three time points. In a study by Keir et al. (6), 83 glioblastoma patients were arbitrarily divided into those who had survived less than or greater than 18 months. Of the LTS, 59% met DT ≥4 cutoff for distress compared with 49% of those diagnosed <18 months ago. This study concluded that regardless of LTS status, distress continued to be a part of the disease trajectory for many glioblastoma patients. This study also concluded that understanding the sources of distress in PBT patients would aid clinical teams in better developing targeted interventions to help address and reduce psychological distress (6).

The longitudinal time course of psychological distress and its relationship to other mood-related symptoms remains to be clarified. Kangas et al. (13) studied the effects of radiotherapy on the psychological [i.e., posttraumatic stress symptoms (PTSS)] and cognitive functioning of adults with PBT, who were assessed at two time points – pre-radiotherapy (T1) and 3.5 months post-radiotherapy (T2). Minimal differences in functioning were found between patients according to BT type (benign *n* = 45 versus malignant *n* = 25). Seventeen percent of the cohort reported clinically elevated PTSS at T1, which reduced to 13% at T2. Younger age (<65 years), reduced quality of life (QoL), and elevated anger symptoms at T1 predicted PTSS at T2, while having a benign BT, low PTSS, and depressive symptoms at T1 were predictive of improved QoL at T2. Lamperti et al. (14) analyzed 81 patients with recurrent central nervous system tumors and found that rather surprisingly the emotional well-being mean score was significantly higher in the recurrence sample than in patients with brain tumors at first diagnosis. Anxiety did not seem to be influenced by a relapse diagnosis; instead, depression was higher and differed significantly from normative data. Their data suggested that some patients retained highly preserved coping strategies for managing emotional distress despite intact judgment and disease awareness.

To our knowledge, the present study has reported DT findings in one of the largest cohorts of non English speaking neuro-oncology populations. Despite this, the current study did not find significant differences in DT scores between non English ­speaking and English speaking groups. It is appreciated that cultural diversity is a much more holistic concept than spoken language alone. Kayser et al. (39) undertook a systematic review of 148 psycho-oncology studies and reported that screening measures of distress had comparable reliability, sensitivity, and specificity for Caucasian, Latino, and Asian samples, but it was unclear if equivalent psychometrics could be found among minority ethnic groups (e.g., African American) and immigrants within countries. Donovan et al. (40) reported on 18 non English translations of the DT and found that although cutoff scores varied by language, country, clinical setting, and sample characteristics, a DT score of 4 maximized sensitivity and specificity. Ongoing research by McGrane et al. (41) in CALD populations has addressed the utility of a culturally competent multilingual unmet needs survey in cancer patients.

Findings from the current study substantiate existing studies reporting that PBT caregivers experience significantly elevated psychological distress (1, 16, 22, 42–44). In the current study, of the 32 caregivers screened at the time of initial PBT diagnosis, 78% met caseness criteria with DT ≥4. Furthermore, 100% (all 14 caregivers) met distress criterion at the time of first tumor recurrence.

Petruzzi et al. (45) assessed patients with the Hospital Anxiety and Depression Scale (HADS), Functional Assessment of Cancer Therapy – Brain (FACT-Br) and caregivers with HADS, Caregiver Reaction Assessment Scale (CRA), and the 36-Item Short-Form Health Survey (SF-36). They reported that most caregivers experienced more depressive and anxiety symptoms compared with patients. In addition, the clinical and psychological features of patients did not correlate with psychological patterns of their own caregivers. In another study by Brown et al. (46) addressing the serial QoL measurements of 197 PBT patients and their caregivers, there was better agreement between patient and caregiver scores when the QOL scores were higher. Such studies underscore the complex interrelationship between the emotional state of the PBT patient and their caregiver over time.

It is therefore relevant to consider potential reasons why caregivers might experience higher levels of psychological distress than patients at the time of diagnosis and at recurrence. Although clinical disclosure of tumor recurrence would always ideally occur in the presence of both the patient and their caregiver/s, it is possible that due to altered recall, insight, or changes in memory and other cognitive processes, the patient is not able to retain all the information and management plans disclosed. In practical terms, it is also not uncommon for caregivers to intentionally seek out additional prognostic information from the treating healthcare team in another confidential forum. Caregivers sometimes prefer to shield the patient from exchanges where poor prognostic news is relayed. Another possible contributing factor to distress is that caregivers may be faced with the additional decisions regarding palliative care options, increasing symptom (physical, cognitive) burden and financial stress.

### Implications of study findings

The results presented here have a number of implications for clinical care of adults with PBT. Firstly, given the elevated levels of psychological distress in this cohort of patients and caregivers, it is imperative that they undergo systematic and routine psychological distress screening. Distress screening is considered a fundamental component of a holistic model for psychological services, which should contribute to the development of a treatment plan and appropriate and timely referrals and thus effective interventions (47).

Due to the projected trajectory of tumor recurrence in HGG patients, such screening, triage, and referral systems need to incorporate repeat screening over time for patients and caregivers at relevant points in the illness trajectory (i.e., diagnosis, recurrence, and the terminal phase). A population of PBT patients and their caregivers would be a potentially ideal group in which to adopt a tiered approach to psychological interventions, as outlined by Hutchison et al. (48), whereby the level of distress and expressed need is matched to an appropriate level of care. Such levels of care range from 1 (universal) for those with minimal or mild distress to supportive (2) and (3) extended care (mild to moderate distress), then increasing to (4) specialized and (5) acute care for those affected by moderate to severe psychological distress.

This then leads to the discussion of the workforce that could provide such care for this particular group of patients and caregivers. In Australia and New Zealand, the majority of cancer care coordinators come from a specialist nurse or allied health professional background (49). Given the rarity of primary malignant brain tumors, with just over 1,700 new projected cases of brain cancer diagnosed in 2014 in Australia, and an incidence of 7.2 per 100,000 (50), a neuro-oncology-specific care coordinator is understandably a very uncommon role and recurrent funding remains an ongoing challenge. The title and the constituents of the role varies considerably, as described in Europe (51–53), North America (26), and other regions such as Israel (54). Some of the roles focus on cancer treatments (such as chemotherapy management) versus a more holistic model of supportive care throughout the entire care trajectory.

A neuro-oncology-specific care coordinator is well positioned to facilitate symptom and needs assessment, psychological support and referrals or intervention throughout the care continuum, and particularly so at predictable time points along the care journey where patients and caregivers are likely to experience higher distress levels. Due to the complexity, this role necessitates an experienced health practitioner familiar with the symptomatology, needs, and journey of a brain tumor patient. It is important that patient and caregiver needs are anticipated, proactively screened for and detected early, to ensure that timely intervention can occur. Furthermore, as many Australian cancer services will not routinely collect nor screen cases such as benign brain tumor patients (as their data are not typically collected by cancer registries), a neuro-oncology service should ideally have mechanisms to support this specific subgroup of PBT patients and their caregivers with a longer survivorship trajectory.

As most cancer services will not have the benefit of a dedicated, neuro-oncology-specific care coordinator, alternate models of service delivery will rely more fully on psycho-oncology staff and/or a programmatic system-wide approach for distress screening (55) and overall coordination of care by all members of the healthcare team. Given the rarity of PBT overall, there have been efforts to expand the knowledge and skillset and educate and train healthcare professionals regarding brain tumors as exemplified by the Australian initiative which included a specific online module about brain cancer (56). Tailored information provision and education is a vital component of the care coordination role (57, 58). In the setting of uncommon tumors, although there are now adequate English language resources covering most aspects of cancer care, there remains a paucity of translated high quality material in non English languages. The costs of translating existing resources remain another practical barrier.

There were several limitations of the current study. Firstly, DT data beyond the first tumor recurrence were not captured, and thus it is possible that patients and/or caregivers could have experienced even higher psychological distress levels during the terminal phase of care. Secondly, not all NESB participants and not all caregivers were able to be assessed – hence, it is unclear to what extent the caregiver sample and NESB sample were representative of the broader population. It is also acknowledged that the caregiver group was relatively small and thus results should ideally be verified in a larger sample. In addition, a notable proportion of benign PBT caregivers were not accessible for assessment due to the fact that benign PBT patients often attended clinical consultations alone.

Finally, it was not possible to compare the level of psychological distress and caseness between the four subgroups due to the differing composition of these groups. Rather, the current study was largely descriptive in summarizing DT data for four different groups in a clinical setting.

Finally, future research directions could include an investigation of the impact of screening for psychological distress at various time points upon the workflow of a cancer care coordinator. It would also be interesting to compare distress levels in patients and caregivers during phases in which there is evidence of disease stability, to better understand the support that is needed.

## Conclusion

This study demonstrates the prevalence of elevated psychological distress in a neuro-oncology population of patients at diagnosis and at first recurrence and also in caregivers. The groups exhibiting the highest distress levels included patients with HGG, patients with disease recurrence, and caregivers. It is thus imperative that both patients and caregivers have access to timely, systematic care coordination and needs assessment as well as a skilled and knowledgeable healthcare team who can provide effective intervention and support across the care trajectory.

## Conflict of Interest Statement

The authors declare that the research was conducted in the absence of any commercial or financial relationships that could be construed as a potential conflict of interest.
